# Longitudinal description and prediction of physical inactivity among patients with borderline personality disorder and personality-disordered comparison subjects

**DOI:** 10.1186/s40479-024-00253-4

**Published:** 2024-06-07

**Authors:** Isabel V. Glass, Frances R. Frankenburg, Mary C. Zanarini

**Affiliations:** 1 Laboratory for the Study of Adult Development, McLean Hospital, 115 Mill Street, Belmont, MA 02478 USA; 2 Boston University School of Medicine, Boston, MA USA; 3 Edith Nourse Rogers Veterans Administration Medical Center, Bedford, MA USA; 4 Harvard Medical School, Boston, MA USA

**Keywords:** Borderline personality disorder, Physical inactivity, Symptomatic and psychosocial recovery, Childhood adversity, Adult adversity, Comorbid medical disorders, Comorbid symptomatic disorders

## Abstract

**Background:**

The physical and psychological benefits of physical activity are well-known, and physical activity has been proven to be a helpful adjunct to psychotherapeutic treatment for many symptomatic disorders, including mood and anxiety disorders. The current study explores physical inactivity levels in patients with borderline personality disorder (BPD). The first aim of this study is to describe the 12-year course of physical inactivity in patients with BPD. The second aim is to examine predictors of physical inactivity, including adversity experiences, comorbid symptomatic (formerly axis I) disorders, medical disorders, and demographic factors.

**Methods:**

Two hundred and forty-five patients with BPD were interviewed seven times over 12-years of prospective follow-up as part of the McLean Study of Adult Development (MSAD). Patients were categorized as ever-recovered (i.e., patient had experienced a symptomatic and psychosocial recovery from BPD) or never-recovered. At each follow-up, patients reported physical activity levels (minutes of exercise per week) via a semi-structured interview— the Medical History and Services Utilization Interview (MHSUI). Data was collected from June 1992 to December 2018.

**Results:**

Never-recovered patients with BPD were significantly more inactive than their ever-recovered counterparts (*p* < 0.001). These rates of inactivity remained stable over time for both groups. Two significant multivariate predictors of inactivity were found: obesity (*p* = 0.003) and PTSD (*p* < 0.001).

**Conclusions:**

Non-recovered BPD patients are more likely to be inactive than patients who have recovered. Both clinical and medical factors appear to contribute to inactivity levels in patients with BPD.

## Introduction

Physical inactivity is a common phenomenon among psychiatric disorders [1], and is particularly concerning due to its association with chronic diseases [2, 3], cardiovascular risk factors [4], and premature mortality [5–7]. Conversely, other studies have shown the positive effect physical activity has on overall wellbeing and psychological health [8–12].

The majority of reviews and studies exploring the relationship between physical activity and psychiatric disorders have focused on mood disorders [13–17], anxiety disorders [18–22], or both [23–25]. These studies have found that physical activity is associated with lower levels of psychiatric symptoms and/or higher levels of overall psychological wellbeing. Despite lack of consensus about optimal frequencies or intensities of physical activity, the basic conclusion is clear: not only does physical activity reduce symptoms of mood [8, 16] and anxiety disorders [19–21, 26], but physical activity also appears to protect against the emergence of such symptoms [21]. Relatedly, several studies have found that physical activity is a helpful adjunct in the treatment of these disorders [8, 19, 26, 27].

Research has also explored the relationship between physical activity and three other major symptomatic disorder (formerly axis I) categories: substance use disorders, post-traumatic stress disorder (PTSD), and eating disorders. Five systematic reviews have assessed the impact of physical activity on substance use, with four reviews finding that physical activity was related to a reduction of substance use behaviors [28–31], in addition to comorbid anxiety and depression symptoms [29, 30]. Interestingly, one study found that though physical activity was a protective factor against drug use in an adolescent population, physical activity actually increased the risk of alcohol use [28]. The final review did not find evidence for a positive effect of physical activity on alcohol usage, but did find relative support for physical activity on global substance use [31]. Similar results have been found for physical activity and PTSD, with metanalyses showing that increasing physical activity is an effective way to decrease PTSD symptoms [32–34] and can be a helpful adjunct in therapeutic treatment of PTSD [35].

Understandably, physical activity and eating disorders is likely the most complex relationship, given that compensatory and/or compulsive exercise is a symptom of many eating disorders [36]. However, of the available literature exploring physical activity and eating disorders, research has indeed suggested that, similar to the other symptomatic disorders reviewed, physical activity can help reduce symptoms [37–40].

With physical activity research primarily focusing on major symptomatic disorder categories (mostly concentrated in mood and anxiety disorders), considerably less attention has been paid to physical inactivity levels in individuals with borderline personality disorder (BPD). Our group published two papers with the first preliminary findings on this relationship, with results showing that non-remitted [41] and non-recovered [42] patients with BPD reported more maladaptive health-related lifestyle choices as compared to their other personality-disordered counterparts, including lack of regular exercise. More recently, St-Amour et al. [43] have explored the potential impact of physical exercise on negative affect in BPD, but found no significant decrease in the level of negative affect in these patients after acute exercise.

The current study aims to explore physical inactivity levels in a large and well-defined sample of patients with BPD over 12 years of prospective follow-up. The descriptive aim of this study is to describe the prevalence rates of physical inactivity in two sub-groups of BPD (ever versus never-recovered) across over a decade of follow-up. The predictive aim of this study is to explore a range of meaningful health-related and clinical predictors of physical inactivity in patients with BPD.

The predictive portion of this paper also seeks to address the literature that has suggested a relationship between childhood adversity and rates of physical (in)activity in adulthood, with associations ranging from weak [44] to strong [45, 46]. It has been well-established that patients with BPD have higher rates of childhood adversity, including experiences of emotional, verbal, physical, and sexual abuse, as well as neglect, compared to other patients with psychiatric disorders and the general public [47–49]. Thus, the predictive aim of this paper will include an exploration of whether childhood (and adult) adversity is a significant predictor of inactivity in adults with BPD when assessed with co-occurring symptomatic disorders, medical conditions, and demographic factors.

## Methods

The current study is part of a multifaceted longitudinal study of the course of borderline personality disorder – the McLean Study of Adult Development (MSAD). The methodology of this study, which was reviewed and approved by the McLean Hospital Institutional Review Board, has been described in detail elsewhere [50]. Briefly, all patients were initially inpatients at McLean Hospital in Belmont, Massachusetts. Each patient was screened to determine that they were 18 to 35 years of age, had a known or estimated IQ of 71 or higher, and had no history or current symptomatology of schizophrenia, schizoaffective disorder, bipolar I disorder, or an organic condition that could cause psychiatric symptoms.

After the study procedures were explained at index admission, written informed consent was obtained. Each patient then met with a master’s-level interviewer blind to the patient’s clinical diagnoses. Three semi-structured diagnostic interviews were administered: the Structured Clinical Interview for DSM-III-R Axis I Disorders (SCID-I) [51], the Revised Diagnostic Interview for Borderlines (DIB-R) [52], and the Diagnostic Interview for DSM-III-R Personality Disorders (DIPD-R) [53]. Good-to-excellent levels of interrater and test-retest reliability were achieved [54, 55].

The Revised Childhood Experiences Questionnaire (CEQ-R), a semi-structured interview, was also administered at index admission [49]. The CEQ-R assess three childhood adversity experiences: sexual abuse, other abuse (i.e., verbal, emotional, and/or physical abuse), and neglect (i.e., physical neglect, emotional withdrawal, inconsistent treatment, denial of patient’s feelings, lack of real relationship with parent or parental figure, caretaker placing patient in parental role, and caretaker’s failure to protect the patient). Each was scored for severity on a continuous scale, resulting in continuous scores for sexual abuse (0–12), other abuse (0–18), and neglect (0–42).

After index admission, patients were re-interviewed 12 times, with each follow-up interview separated by two years. At follow-up waves, diagnostic information was assessed via the same three diagnostic interviews used at study entrance. After informed consent was obtained, the MSAD diagnostic battery was re-administered. Good-to-excellent interrater reliability was maintained throughout the study for both symptomatic and personality disorder diagnoses [54, 55].

In addition to the core diagnostic battery, two additional semi-structured interviews were administered at each follow-up wave starting at two-year follow-up. The Revised Borderline Follow-up Interview (BFI-R) [56], which is the longitudinal analog to our baseline measure of demographics, psychosocial functioning, and treatment history (The Background Information Schedule [BIS]), assessed social and vocational functioning at all follow-up periods, including socioeconomic status (SES), education level and usage of social security insurance (i.e., SSDI). The Follow-Up Version of the Abuse History Interview (AHI) [57], similar to the BFI-R, is the longitudinal analog to our baseline measure of abuse (Abuse History Interview [AHI]). Both the AHI and its follow-up analog assessed adult experiences of violence, including verbal, emotional, physical, and sexual abuse at each follow-up period of the study. The AHI has been shown to have good psychometric properties [58].

Beginning at six-year follow-up through the end of the study, the Medical History and Services Utilization Interview (MHSUI) was administered to all patients. The MHSUI assesses physical health, lifestyle issues related to physical health (including questions about regular exercise), and health care utilization [41]. Medical diagnoses were not recorded unless they had been given to the subject by a physician.

In this study, physical inactivity was determined using the MHSUI interview, based on the question: “Do you exercise regularly? If so, how many minutes per week?” Participants were asked to provide an approximated time spent on physical activity during a typical week. A subject was considered inactive on the MHSUI if they reported less than 150 min per week of exercise. This definition mirrors general national guidelines for physical activity [59].

Though the MHSUI was first administered at the six-year follow-up, the latter part of the question probing for minutes of exercise per week was only added at the 12-year follow-up wave (i.e., early to mid-2000s), which coincided with the CDC updating their guidelines in 2008 to 150 min of exercise per week for an average adult [59]. As such, the term ‘baseline’ will refer to the 12-year follow-up, as that was the first wave that our definition of inactivity (using minutes/week) was measured.

### Definition of recovery

Our measure of recovery had three elements. Recovery elements included: (1) a concurrent symptomatic remission from BPD (defined as no longer meeting DIB-R and DSM-III-R criteria for BPD for a period of at least two years), (2) having at least one emotionally sustaining relationship with a close friend or life partner or spouse, and (3) being able to work or go to school consistently, competently, and on a full-time basis.

Recovery status was assessed at each two-year follow-up via the BFI-R as well as the DIB-R and DSM-III-R criteria sets for BPD. Participants who recovered from BPD during the 12-year longitudinal study period were categorized as “ever-recovered” and those who did not were categorized as “never-recovered.”

### Statistical analyses

Generalized estimating equations (GEE), with recovery status and time as main effects of interest, were used in longitudinal analyses of prevalence data. A test of recovery status by time interaction was also conducted. The inclusion of the recovery status by time interaction term allowed for a comparison of the patterns of change in inactivity over time between groups. However, this interaction term was not significant, and was thus removed from final analyses. The estimated coefficients of the GEE analyses, when exponentiated, yielded relative differences for recovery status and time. The p-level was set at *p* < 0.05 for the between-group comparison of inactivity.

GEE analyses were also used to examine predictors of inactivity in patients with BPD. Predictors represent static variables measured at index admission (CEQ-R) or study baseline (i.e., 12-year follow-up) (AHI, BFI-R, MHSUI, SCID). Predictors include childhood adversity experiences (severity of sexual abuse, severity of other abuse, severity of neglect), adult adversity experiences (physical partner violence, rape history), medical conditions (osteoarthritis, chronic obstructive pulmonary disease [COPD], and obesity), comorbid symptomatic disorders (major depression, any anxiety disorder, any substance use disorder, any eating disorder, or PTSD within the last two years), and demographic factors (sex, SES, college education, and SSDI utilization). Regarding the symptomatic disorders, any anxiety disorder included panic disorder, agoraphobia, social phobia, simple phobia, obsessive-compulsive disorder (OCD), and generalized anxiety disorder (GAD), but excluded PTSD, as PTSD was classified as a trauma-spectrum disorder. Any substance disorder included alcohol and drug use disorder. Any eating disorder included anorexia nervosa, bulimia nervosa, and eating disorder not otherwise specified (EDNOS), which included binge eating disorder.

All significant bivariate predictors with a p-level of < 0.05 were put into a multivariate model. To select the subset of predictors to be retained in the most parsimonious multivariate model, we compared a sequence of models in a backwards deletion manner. In both the bivariate and multivariate models, assessment period was always included as a covariate to allow for temporal variation in physical inactivity. The significance level of the multivariate model was set at *p* < 0.01.

## Results

### Participants

Two hundred and forty-five patients with BPD were interviewed at 12-year follow-up, when the version of the MHSUI containing the question regarding minutes of exercise per week was first administered. The demographic characteristics of these subjects are detailed in Table 1. Briefly, ever-recovered patients with BPD were significantly younger than never-recovered BPD subjects. There were no significant between-group differences for sex or race.

**Table 1:** Demographics characteristics of ever-recovered and never-recovered patients with borderline personality disorder

	Ever-Recovered (*N* = 148)		Never-Recovered (*N* = 97)		Ever-Recovered vs. Never-Recovered	
	%	*N*	%	*N*	χ²	*p*-value
Female	84.5	125	76.3	74	2.57	0.109
Nonwhite	14.2	21	9.3	9	1.32	0.251
	**M**	**SD**	**M**	**SD**	**t-test**	**p-value**
Age	37.5	5.8	40.5	5.5	3.98	< 0.001*

Attrition was low throughout the study, with 206/249 (83%) of surviving patients with BPD (15 died by suicide and 26 died of other causes) remaining in the study through all 24 years of prospective follow-up.

### Physical inactivity

Table 2 details the prevalence rates of physical inactivity among ever- and never-recovered BPD patients. Approximately 71% of never-recovered BPD patients reported being physically inactive at baseline (i.e., 12-year follow-up), as compared to 56% of ever-recovered patients. By 24-year follow-up, these prevalence rates held steady for the recovered group (approximately 56%), while decreasing to approximately 61% for the non-recovered group. Results from the regression analyses are displayed in the final three columns of Table 2. Age was added as a covariate in this model for recovery status due to the significant between-group difference (see Table 1 for details). Results can be interpreted in terms of relative differences between recovery status and relative change in inactivity levels over time. For instance, never-recovered patients who never recovered from BPD were 15% ([1-0.85]x100%) more likely to report being physically inactive than their recovered counterparts. In terms of relative change over time, inactivity rates for both groups taken together had an insignificant change over time, indicating that inactivity rates remained stable for both groups.

**Table 2:** Longitudinal prevalence rates of inactivity (%, N) among ever-recovered and never-recovered patients with BPD

	Follow-Up Period							Rel. Diff.* Diagnosis Time	95% CI Diagnosis Time	P-value Diagnosis Time
	12 YR FU	14 YR FU	16 YR FU	18 YR FU	20 YR FU	22 YR FU	24 YR FU			
**Recovered**	56.1 (83)	54.5 (79)	50.4 (71)	44.2 (61)	55.8 (77)	51.5 (70)	55.5 (57)	0.85 0.98	0.79, 0.92 0.92, 1.05	< 0.001* 0.639
**Non-Recovered**	71.1 (69)	70.2 (66)	73.6 (67)	69.0 (60)	71.8 (61)	77.2 (61)	61.0 (47)			

Note: Analyses are controlled for age

*Relative difference = (1 – RR) x 100%

### Predictor analyses

Table 3 displays the mean values and prevalence rates of the 17 predictor variables. These include three childhood abuse variables (CEQ-R), two adult abuse variables (AHI), three medical conditions (MHSUI), five symptomatic disorders (SCID), and four demographic factors (BFI-R).

**Table 3:** Descriptive characteristics of predictor variables

	Mean	SD
**Childhood Adversity**		
Severity of Sexual Abuse	1.8	2.04
Severity of Other Abuse	7.2	5.25
Severity of Neglect	14.4	10.25
	**%**	**N**
**Adult Adversity**		
Physical Partner Violence	1.6	4
Rape History [a]	4.1	10
**Medical Conditions**		
Osteoarthritis	9.4	23
COPD	2.5	6
Obesity	30.2	74
**Symptomatic Disorders**		
Major Depression	45.7	112
Any Anxiety Disorder [b]	40.0	98
Any Substance Use Disorder [c]	12.7	31
PTSD	19.2	47
Any Eating Disorder [d]	19.6	48
**Demographic Factors**		
Sex (Female)	81.2	199
Socioeconomic Status [e]	3.3 (M)	1.5 (SD)
College Education	49.8	122
SSDI Utilization	44.5	109

a Includes sexual abuse by a caretaker or non-caretaker

b Includes all anxiety disorders (panic disorder, agoraphobia, social and simple phobias, OCD and GAD)

c Includes alcohol and drug use disorder

d Includes anorexia nervosa, bulimia nervosa, and EDNOS (which includes binge eating disorder)

e Based on the Hollingshead Index, where 1 = lowest and 5 = highest [75]

Table 4 displays results of the bivariate predictor analyses. Two childhood adversity experiences (severity of sexual abuse, severity of neglect), one adult adversity experience (rape history), three medical conditions (osteoarthritis, COPD, obesity), one symptomatic disorder (PTSD), and three demographic factors (socioeconomic status, college education, and SSDI utilization) were significant bivariate predictors of inactivity. In the multivariate model using backwards deletion (Table 5), only one medical condition (obesity) and one symptomatic disorder (PTSD) remained significant, with obesity increasing one’s risk of being physical inactive by 13% and PTSD increasing the risk by 17% when studied together.

**Table 4:** Bivariate predictors of physical inactivity in patients with BPD

	Coefficient	*p*-value	95% CI
**Childhood Adversity**			
Severity of Sexual Abuse	1.03	0.005*	1.01, 1.04
Severity of Other Abuse	1.01	0.116	1.00, 1.01
Severity of Neglect	1.00	0.032*	1.00, 1.01
**Adult Adversity**			
Physical Partner Violence	1.03	0.548	0.93, 1.14
Rape History	1.21	0.003*	1.07, 1.38
**Medical Conditions**			
Osteoarthritis	1.16	0.012*	(1.03, 1.31)
COPD	1.35	< 0.001*	(1.19, 1.52)
Obesity	1.14	0.002*	(1.05, 1.24)
**Symptomatic Disorders**			
Major Depression	1.01	0.903	(0.93, 1.09)
Any Anxiety Disorder	1.05	0.218	(0.97, 1.14)
Any Substance Use Disorder	1.05	0.327	(0.95, 1.15)
PTSD	1.18	< 0.001*	(1.09, 1.28)
Any Eating Disorder	1.01	0.806	(0.92, 1.12)
**Demographic Factors**			
Sex	0.95	0.357	(0.86, 1.05)
Socioeconomic Status	1.04	0.002*	(1.02, 1.07)
College Education	0.90	0.006*	(0.83, 0.97)
SSDI Utilization	1.15	< 0.001*	(1.07, 1.25)

**Table 5:** Multivariate predictors of physical inactivity in patients with BPD

	Coefficient	*p*-value	95% CI
**Medical Conditions**			
Obesity	1.13	0.003*	(1.04, 1.23)
**Symptomatic Disorders**			
PTSD	1.17	< 0.001*	(1.08, 1.27)

## Discussion

Three important findings have emerged. First, never-recovered BPD patients were significantly more likely to report being physically inactive than ever-recovered patients. This finding is relatively unsurprising, as this may be part of the pattern of unhealthy lifestyle choices in the non-remitted or non-recovered group that we have detailed before, including smoking, alcohol use, daily sleep medication use, and use of pain medications [41, 42]. Conversely, these findings also suggest that achieving a symptomatic and psychosocial recovery from BPD may positively influence rates of physical activity (i.e., increased movement or activity due to gainful employment, enrollment in full-time classes, a more robust social life, etc.)

Our second finding is that, taken together, rates of inactivity for both ever- and never-recovered patients remained stable over time. This finding is both relatively surprising and unsurprising, given that one might expect inactivity to increase over time due to age-related conditions that could make physical activity more challenging (e.g., osteoarthritis). Alternatively, one might have even expected inactivity levels to decrease over the decade of follow-up in this study, given shifting cultural attitudes and/or behaviors related to physical activity, as epidemiological studies have shown that people are more active now than they were a decade ago [60–63]. Ultimately, however, both groups’ inactivity levels remained stable over time. Moreover, even though ever-recovered patients had significantly lower levels of inactivity compared to their never-recovered counterparts, it is important to note that still over half (56%) of ever-recovered patients reported being inactive by 24-year follow-up. These rates of inactivity are approximately twice that of the national American average, which was reported to be approximately 25% in 2022 [59]. This ultimately suggests that though recovery from BPD may be associated with lower levels of inactivity, physical inactivity still remains a problem for many of those with BPD.

Our third finding revealed two significant multivariate predictors of inactivity: obesity and PTSD. Both were positively associated with inactivity, with a diagnosis of obesity increasing the risk of inactivity by 13% and PTSD by 17% when studied together. This model suggests that an interaction between PTSD and obesity may be driving the higher levels of inactivity in patients with BPD. Prior research has shown that patients with PTSD report low levels of physical activity [32, 34, 64–66] and higher rates of obesity [65–69], which may go on to interact with BPD.

Despite some being significant bivariate predictors, none of the childhood or adult adversity variables were significant multivariate predictors. This suggests that inactivity in adult patients with BPD is driven far more by current medical and psychological problems rather than adversity experiences from either childhood or adulthood. However, it could be that the PTSD (which was a significant multivariate predictor) is directly related to these adversity experiences (such as severity of sexual abuse, severity of neglect, and history of rape, all of which were significant bivariate predictors of inactivity in patients with BPD).

### Clinical implications

The psychological problems of patients with BPD may be overwhelming and distract the clinician from realizing the benefits, both medical and psychological, that can come from physical activity. The initiation of physical activity, even in middle-aged and older men, or in people who are medically compromised, has been associated with decreased mortality [70, 71]. Moreover, not only does this intervention decrease mortality, but it is associated with increased quality of life with minimal or few adverse effects [70]. Whether this would be true with respect to the symptoms of BPD has not yet been established but is worth exploring.

The causal relationship between physical inactivity and the persistence of borderline personality cannot be established in this study. But there is enough evidence to show that physical activity is helpful for alleviating depression and anxiety, common problems in people with BPD, that it should be encouraged.

Mental health clinicians are uniquely positioned to help their patients with BPD understand the benefits of physical activity, as they see them on a far more frequent basis than their primary care physicians. Clinicians can help their patients to understand and overcome perceived barriers to activity and explore their preferences and/or concerns related to physical activity. Physical activity should be included on the list of self-care behaviors that clinicians routinely encourage.

### Limitations

The current study has several limitations. The first limitation is that patients were all inpatients at the time of index admission. It may be that levels of physical inactivity are different in outpatients or non-patients with BPD. An additional limitation is that the majority of patients in this study also participated in some type of intermittent treatment as usual in the community (including individual psychotherapy and/or psychotropic medication) during the course of the study [72, 73]. Thus, it is unknown whether inactivity levels may be related to the typically intermittent non-evidenced based treatment those with BPD were receiving in the community.

A third limitation of our study is related to our definition of physical activity. We were unable to differentiate between how many minutes patients spent engaging in moderate and vigorous activity in this study. Though the most promulgated CDC guideline highlights 150 min of moderate activity [59], the guidelines alternatively advise 75 min of vigorous activity, or some combination of the two. Although we collected information regarding the types of exercise patients engaged in, the vast majority endorsed two or more forms of activity in their daily lives, and we did not individually record the minutes spent on each type. Thus, we were not able to retrospectively ascertain more granular information regarding minutes at different intensity levels. Future studies would benefit from detailing the number of minutes spent in moderate and vigorous exercise, which would have given a more granular assessment of levels of activity.

Another possible limitation is that the data was obtained by self-report. However, research from federal surveys has shown that there is acceptable-to-good concordance with respect to physical health when self-report and medical records or physical measures are compared [74].

Future studies should include quantified descriptions of physical activity (which our lab is now pursuing), exploration of possible mechanisms mediating decrease in symptoms, and more precise measures of physical fitness. Psychosocial environmental factors, such as the availability of safe places to exercise, and cultural attitudes towards physical activity could also be explored.

## Conclusions

In sum, we found that never-recovered patients with BPD reported higher levels of inactivity than their ever-recovered peers. These rates of inactivity remained stable across 12-years of follow-up. Both co-occurring symptomatic disorders (PTSD) and medical factors (obesity) appear to play a role in levels of inactivity in patients with BPD.

## Data Availability

Requests for data must be made to Dr. Mary C. Zanarini. Data is held in a secure cloud at Mass General Brigham, McLean’s parent company.

## Abbreviations

AHI: Abuse history interview
BPD: Borderline personality disorder
COPD: Chronic obstructive pulmonary disease
DIPD-R: Diagnostic interview for DSM-III-R personality disorders
EDNOS: Eating disorder not otherwise specified
GAD: Generalized anxiety disorder
GEE: Generalized estimating equations
MHSUI: Medical history and services utilization interview
MSAD: McLean study of adult development
OCD: Obsessive-compulsive disorder
PTSD: Post-traumatic stress disorder
BFI-R: Revised borderline follow-up interview
CEQ-R: Revised childhood experiences questionnaire
DIB-R: Revised diagnostic interview for borderlines
SSDI: Social security disability insurance
SES: Socioeconomic status
SCID-I: Structured clinical interview for DSM-III-R Axis I disorders
